# Supplementary material for: The effect of cumulative ecological risk on migrant children’s Internet game addiction: a moderated mediation model
Source: PeerJ. 2025 Aug 28;13:e19787. doi: 10.7717/peerj.19787 (PMC12399076; doi:10.7717/peerj.19787)
Supplement: Supplemental Information 3 [file peerj-13-19787-s003.doc]

亲爱的同学：

您好！感谢您抽出时间配合本次调查。请阅读下面各题，每题**只能选择** **1个**最适合自己的打√ , 答案没有好坏之分。本调查采用不记名的形式，我们将对您的回答严格保密。希望能够得到您的支持。

**1 你的性别：**①男 ②女

**2 年级：** ①初一 ②初二 ③初三

**3** 你的年龄 岁

**4 我是否从出生就在这：**①是 ② 否，我从农村搬迁至此地

**5 我居住在这里的时间：**

① 从出生就在 ② 半年以下 ③ 半年至一年 ④ 1-2 年 ⑤ 2年以上

**6 您父亲文化水平：**

①没有上过学 ②小学 ③初中 ④高中或中专 ⑤大学（专科或本科） ⑥研究生

**7 您母亲文化水平：**

①没有上过学 ②小学 ③初中 ④高中或中专 ⑤大学（专科或本科） ⑥研究生

**8 我希望自己的学业达到什么水平：**

①初中毕业 ②高中 (含职高或中专) ③大学(大专或本科) ④硕士 ⑤博士

| **题** **号** | **题目** | **非** **常** **不** **符** **合** | **有** **点** **不** **符** **合** | **不清楚** | **有** **些** **符** **合** | **非** **常** **符** **合** |
| --- | --- | --- | --- | --- | --- | --- |
| 1 | 我家没有足够的钱买新衣服 |  |  |  |  |  |
| 2 | 我家没有足够的钱买我喜欢的食物 |  |  |  |  |  |
| 3 | 我家没有足够的钱买好的住房 |  |  |  |  |  |
| 4 | 我家没有剩余的钱供一家人去娱乐 |  |  |  |  |  |
| 5 | 我的父亲和母亲之间的关系很好 |  |  |  |  |  |
| 6 | 你的父亲和母亲常吵架 |  |  |  |  |  |
| 7 | 我觉得我和我们学校里的人关系亲近 |  |  |  |  |  |
| 8 | 我觉得我很高兴成为学校的一份子 |  |  |  |  |  |
| 9 | 我认为我在学校是开心的 |  |  |  |  |  |
| 10 | 学校我能得到公平的对待 |  |  |  |  |  |
| 11 | 我在学校里感到安全 |  |  |  |  |  |
| 12 | 我很在意自己的网络游戏行为 |  |  |  |  |  |
| 13 | 我需要用更多的时间来玩网络游戏以获得满足或者开心 |  |  |  |  |  |
| 14 | 因为玩网游而对以前的爱好和其他娱乐活动失去了兴趣 |  |  |  |  |  |
| 15 | 尽管游戏会在我和其他人（如父母）造成问题，我也继续玩游戏 |  |  |  |  |  |
| 16 | 我玩网络游戏是为了逃避或缓解负面的情绪 |  |  |  |  |  |
| 17 | 我因为网游而影响或者失去了一段关系或是学习的机会 |  |  |  |  |  |
| 18 | 我会想方法减少玩网络游戏的时间但最终失败 |  |  |  |  |  |
| 19 | 我会对人隐瞒你玩网络游戏的时长 |  |  |  |  |  |
| 20 | 我会因为不能玩网络游戏而变得很烦躁 |  |  |  |  |  |

| **1 业余时间你与这个人在一起的时间有多长？** | | | | | |
| --- | --- | --- | --- | --- | --- |
|  | **从不** | **很少** | **有时** | **很多** | **几乎总是** |
| **父母** |  |  |  |  |  |
| **老师** |  |  |  |  |  |
| **朋友** |  |  |  |  |  |
| **2 你与这个人在一起时会感到烦恼吗?** | | | | | |
|  | **从不** | **很少** | **有时** | **很多** | **几乎总是** |
| **父母** |  |  |  |  |  |
| **老师** |  |  |  |  |  |
| **朋友** |  |  |  |  |  |
| **3 这个人会教你做你不会做的事吗?** | | | | | |
|  | **从不** | **很少** | **有时** | **很多** | **几乎总是** |
| **父母** |  |  |  |  |  |
| **老师** |  |  |  |  |  |
| **朋友** |  |  |  |  |  |
| **4 你与这个人的关系好吗?** | | | | | |
|  | **一点也不好** | **有点好** | **比较好** | **很好** | **非常好** |
| **父母** |  |  |  |  |  |
| **老师** |  |  |  |  |  |
| **朋友** |  |  |  |  |  |
| **5 你会告诉这个人各种事情吗？** | | | | | |
|  | **从不** | **很少** | **有时** | **很多** | **几乎总是** |
| **父母** |  |  |  |  |  |
| **老师** |  |  |  |  |  |
| **朋友** |  |  |  |  |  |
| **6 这个人喜欢你或者爱你吗？** | | | | | |
|  | **一点也不喜欢** | **有点喜欢** | **比较喜欢** | **很喜欢** | **非常喜欢** |
| **父母** |  |  |  |  |  |
| **父亲** |  |  |  |  |  |
| **老师** |  |  |  |  |  |
| **朋友** |  |  |  |  |  |
| **7 这个人经常惩罚你吗？** | | | | | |
|  | **从不** | **很少** | **有时** | **很多** | **几乎总是** |
| **父母** |  |  |  |  |  |
| **老师** |  |  |  |  |  |
| **朋友** |  |  |  |  |  |
| **8 与这个人在一起时，你感到受尊重吗？** | | | | | |
|  | **从不** | **很少** | **有时** | **很多** | **几乎总是** |
| **父母** |  |  |  |  |  |
| **老师** |  |  |  |  |  |
| **朋友** |  |  |  |  |  |
| **9 你与这个人在一起玩或娱乐的时间有多少？** | | | | | |
|  | **从不** | **很少** | **有时** | **很多** | **几乎总是** |
| **父母** |  |  |  |  |  |
| **老师** |  |  |  |  |  |
| **朋友** |  |  |  |  |  |
| **10 你与这个人经常看法不一致并争吵吗？** | | | | | |
|  | **从不** | **很少** | **有时** | **很多** | **几乎总是** |
| **父母** |  |  |  |  |  |
| **老师** |  |  |  |  |  |
| **朋友** |  |  |  |  |  |
| **11 当你遇到问题时，这个人会帮你解决吗？** | | | | | |
|  | **从不** | **很少** | **有时** | **很多** | **几乎总是** |
| **父母** |  |  |  |  |  |
| **老师** |  |  |  |  |  |
| **朋友** |  |  |  |  |  |
| **12 你与这个人一起做事的方式使你感到愉快吗？** | | | | | |
|  | **一点也不愉快** | **有点愉快** | **比较愉快** | **很愉快** | **非常愉快** |
| **父母** |  |  |  |  |  |
| **老师** |  |  |  |  |  |
| **朋友** |  |  |  |  |  |
| **13 你会把内心秘密告诉这个人吗？** | | | | | |
|  | **从不** | **很少** | **有时** | **很多** | **几乎总是** |
| **父母** |  |  |  |  |  |
| **老师** |  |  |  |  |  |
| **朋友** |  |  |  |  |  |

| **14 这个人真的很关心你吗？** | | | | | |
| --- | --- | --- | --- | --- | --- |
|  | **从不** | **很少** | **有时** | **很多** | **几乎总是** |
| **父母** |  |  |  |  |  |
| **老师** |  |  |  |  |  |
| **朋友** |  |  |  |  |  |
| **15 这个人会由于你不服从他（她）而约束训斥你吗？** | | | | | |
|  | **从不** | **很少** | **有时** | **很多** | **几乎总是** |
| **父母** |  |  |  |  |  |
| **老师** |  |  |  |  |  |
| **朋友** |  |  |  |  |  |
| **16 这个人认为你很能干** | | | | | |
|  | **从不** | **很少** | **有时** | **很多** | **几乎总是** |
| **父母** |  |  |  |  |  |
| **老师** |  |  |  |  |  |
| **朋友** |  |  |  |  |  |
| **17 你和这个人经常一起出去并做一些开心的事吗？** | | | | | |
|  | **从不** | **很少** | **有时** | **很多** | **几乎总是** |
| **父母** |  |  |  |  |  |
| **老师** |  |  |  |  |  |
| **朋友** |  |  |  |  |  |
| **18 你和这个人经常互相争论吗？** | | | | | |
|  | **从不** | **很少** | **有时** | **很多** | **几乎总是** |
| **父母** |  |  |  |  |  |
| **老师** |  |  |  |  |  |
| **朋友** |  |  |  |  |  |
| **19 当你需要做事情时这个人会帮助你吗？** | | | | | |
|  | **从不** | **很少** | **有时** | **很多** | **几乎总是** |
| **父母** |  |  |  |  |  |
| **老师** |  |  |  |  |  |
| **朋友** |  |  |  |  |  |
| **20 你对这个人的关系感到满意吗？** | | | | | |
|  | **一点也不满意** | **有点满意** | **比较满意** | **很满意** | **非常满意** |
| **父母** |  |  |  |  |  |
| **老师** |  |  |  |  |  |
| **朋友** |  |  |  |  |  |
| **21 你会把秘密告诉这个人吗？** | | | | | |
|  | **从不** | **很少** | **有时** | **很多** | **几乎总是** |
| **父母** |  |  |  |  |  |
| **老师** |  |  |  |  |  |
| **朋友** |  |  |  |  |  |
| **22 这个人对你有很深厚的情感吗？** | | | | | |
|  | **从不** | **很少** | **有时** | **很多** | **几乎总是** |
| **父母** |  |  |  |  |  |
| **老师** |  |  |  |  |  |
| **朋友** |  |  |  |  |  |
| **23 当你做了不应该做的事时，这个人会责备我吗？** | | | | | |
|  | **从不** | **很少** | **有时** | **很多** | **几乎总是** |
| **父母** |  |  |  |  |  |
| **老师** |  |  |  |  |  |
| **朋友** |  |  |  |  |  |

| **24 这个人会喜欢或称赞我做的事情** | | | | | |
| --- | --- | --- | --- | --- | --- |
|  | **从不** | **很少** | **有时** | **很多** | **几乎总是** |
| **父母** |  |  |  |  |  |
| **老师** |  |  |  |  |  |
| **朋友** |  |  |  |  |  |

**如果您有任何疑问，可与班级老师或项目负责人联系。**

**负责人：林铮铮**

**电话：13123082156**
